# Do patients with fibromyalgia syndrome and healthy people differ in their opinions on placebo effects in routine medical care?

**DOI:** 10.1111/papr.70000

**Published:** 2025-01-27

**Authors:** Johan P. A. van Lennep, Simone Meijer, Merve Karacaoglu, Ralph Rippe, Kaya J. Peerdeman, Henriët van Middendorp, Andrea W. M. Evers

**Affiliations:** ^1^ Health, Medical and Neuropsychology Unit, Faculty of Social and Behavioural Sciences Leiden University Leiden The Netherlands; ^2^ The Center for Interdisciplinary Placebo Studies Leiden Leiden The Netherlands; ^3^ Department of Methodology and Statistics, Faculty of Social Sciences Leiden University Leiden The Netherlands; ^4^ Department of Psychiatry Leiden University Medical Center Leiden The Netherlands; ^5^ Medical Delta, Leiden University, Technical University Delft, and Erasmus University Leiden The Netherlands

**Keywords:** behavioral medicine, fibromyalgia

## Abstract

**Objectives:**

Placebo effects can relieve acute and chronic pain in both research and clinical treatments by learning mechanisms. However, the application of placebo‐based treatment strategies in routine medical care is questioned. The current study investigated the opinions of patients with fibromyalgia and healthy controls regarding learning of placebo effects and their practical applications.

**Method:**

An online survey asked 158 age‐ and sex‐matched adult patients and controls (79 per group) to rate the perceived influence of various placebo learning mechanisms on pain relief, and the acceptability and perceived effectiveness of placebo‐based strategies (open‐label, closed‐label, dose‐extending, and treatment‐enhancing strategies). Respondents' knowledge about placebo effects was obtained through a 7‐item quiz.

**Results:**

The groups did not differ in the perceived influence of placebo learning mechanisms on pain relief (*p* = 0.217). Controls considered closed‐label and treatment‐enhancing strategies more acceptable than patients (*p* = 0.003 and *p* < 0.001), whereas controls perceived all strategies more effective. In both groups, closed‐label strategies were significantly less acceptable than any other strategy (*p*‐values < 0.001), and treatment‐enhancing or dose‐extending strategies were most acceptable. Higher acceptability was predicted by higher perceived effectiveness ratings (*p* < 0.001). Also, increased placebo knowledge was related to higher acceptability (*p* = 0.03) and perceived effectiveness (*p* < 0.001).

**Discussion:**

This survey suggests that both the medical history of patients and knowledge about placebo effects affect the acceptability and perceived effectiveness of placebo‐based strategies. Furthermore, strategies that are transparent, assumed effective, or combined with existing medical treatments are deemed most acceptable. Keeping these factors in mind is essential for the clinical implementation of placebo‐based strategies in routine medical care.

## INTRODUCTION

The psychosocial context surrounding a medical treatment can evoke beneficial effects that are not attributable to active treatment components, also known as placebo effects.^1^, ^2^, ^3^ Inducing these effects by learning mechanisms (eg, instructional learning, associative learning, and social learning) in research settings, is known to relieve clinical symptoms, especially pain (either acute, or chronic).^4^, ^5^, ^6^ However, their clinical implementation, for instance, in pain management, is hampered by ethical concerns involving the possible required deception.^7^ As placebo effects have traditionally been studied by administering inert substances to participants without informing them (ie, deceptive or closed‐label placebos), their use in clinical practice remained controversial. More ethical treatment strategies to evoke placebo effects have resultantly been studied, such as non‐deceptive (open‐label) strategies,^8^ treatment‐enhancing (add‐on) strategies,^9^ or dose‐extending strategies.^10^ In open‐label placebo strategies, the recipient of a placebo is made aware that he/she is receiving an inert substance, yet they could experience somatic effects due to their intrinsic expectations.^11^ When applying treatment‐enhancing strategies, a caretaker tries to boost placebo effects that exist along an established medical treatment by adding an inert substance.^1^ Dose‐extending placebo strategies aim to use placebo effects to substitute an existing pharmaceutical regimen with inert medicine to reduce the amount of actual medicine usage.^10^ Although these strategies have been proven effective in relieving pain symptoms,^10^, ^12^, ^13^ their successful implementation also relies on the opinions and attitudes of the ones receiving them. Studying moral standpoints (eg, acceptability), beliefs (eg, perceived effectiveness), knowledge, and medical history (eg, patients versus healthy individuals) of recipients aids to the implementation process as these factors can influence a patient's treatment adherence. However, the insights into the opinions and attitudes of recipients regarding placebo effects, and strategies based upon them, are limited to a handful of previously conducted cross‐sectional studies.^5^, ^9^, ^14^, ^15^, ^16^, ^17^, ^18^

These studies have indicated that the acceptability of placebo‐based strategies depends on the involved deception and/or the expected outcome (ie, perceived effectiveness).^19^, ^20^, ^21^ However, these surveys did not assess recipients' views of the underlying learning mechanisms of placebo‐based strategies, which create their inherent treatment effectiveness.^18^, ^19^, ^20^, ^21^, ^22^ Also, the influence of medical history on (the application of) placebo‐based strategies, by comparing healthy individuals and patients directly, was rarely taken into account.^23^ A single cross‐sectional study reported that patients with depression less accepted placebo‐based strategies aimed at the treatment of their own disease symptoms than healthy individuals.^24^ Whether these findings generalize to other patient groups and symptoms beyond one's own condition remains to be examined.^24^ It might be particularly relevant to study opinions about placebo effects and their implementation in clinical care in patients with chronic pain, especially in patients with fibromyalgia. First of all, the effect of placebos is more established in pain compared with depression.^25^ Second, chronic pain interacts with someone's emotions, cognitions, and even personality and can thus influence opinions or attitudes toward treatments or strategies.^26^, ^27^ Finally, patients with fibromyalgia are often subjected to a vast amount of ineffective clinical treatments and subsequent encounters because of the difficulty of treating their symptoms. As such, they often experience feelings of invalidation or skepticism toward treatments or treatment providers, and these could be particularly relevant opinions for implementing placebo‐based treatment strategies. A study that encompasses all the above‐mentioned constructs (underlying learning mechanisms, acceptability, perceived effectiveness, knowledge, and medical history) could therefore help to identify what placebo‐based strategies are most suited for clinical applications.

In this study, we investigated the difference in opinions between patients with fibromyalgia and healthy controls regarding the perceived influence of placebo learning mechanisms on pain relief during routine medical care. Additionally, the acceptability and perceived effectiveness of placebo‐based strategies, their acceptability in case of different symptoms (eg, pain or insomnia), and overall knowledge about placebo effects were studied. Lastly, any relationships between acceptability, perceived effectiveness, and knowledge were explored.^21^ We hypothesized that patients would perceive the influence of placebo learning mechanisms to be different from that of healthy controls, mainly because patients with chronic pain are more skeptical than healthy controls toward caregivers, due to invalidation of symptoms, or toward previously experienced ineffective treatments.^28^ For the placebo‐based strategies, we hypothesized that patients would consider them less acceptable and effective than healthy controls, especially in disease‐relatable symptoms (ie, chronic pain, psychological symptoms), following the results of a previous survey in patients with depression.^24^ Furthermore, we hypothesized that strategies that were more known to their recipients, contained minimal deception (ie, open‐label), or with higher expected effectiveness, would be considered more acceptable than others in both groups.^18^, ^29^

## METHODS

This study was part of a larger survey on attitudes and opinions toward placebo and nocebo effects conducted at the Department of Health, Medical, and Neuropsychology, Faculty of Social and Behavioral Sciences of Leiden University in the Netherlands. Nocebo effects are the counterpart of placebo effects and essentially harmful psychosomatic effects due to the psychosocial context surrounding a medical treatment.^30^ However, as the current sub‐study was focused on placebo effects, the nocebo items from the larger survey were not included in the analysis of the current article. Ethical permission for the protocol was granted by the Leiden University Psychology Research Ethics Committee (2021‐04‐15‐A.W.M.Evers‐V3‐3166). The results were reported following The Strengthening the Reporting of Observational Studies in Epidemiology (STROBE) Statement: guidelines for reporting observational studies.^31^

### Respondents

Respondents were recruited between April 2021 and November 2022 through online advertisement (eg, via social media such as Facebook groups, websites of patient organizations, or other online platforms such as SONA) or direct contact. Eligible respondents had to be either patients with fibromyalgia syndrome (FMS) or healthy controls, older than 18 years, able to understand written or spoken Dutch, and have access to a computer with internet connection. Patients with FMS also required a (self‐reported) clinical diagnosis from a general practitioner or medical specialist (eg, rheumatologist). Patients with FMS were ineligible if they had a severe somatic or psychological co‐morbidity (eg, cancer, rheumatic condition, schizophrenia, or PTSD), with the exception of anxiety, depression, sleep disturbances, or other chronic pain symptoms. Healthy controls were ineligible if they had a co‐morbidity that was either (1) being actively treated but still led to one symptom per month, or (2) not treated but led to a symptom more than once a week, or (3) constituted pain symptoms.

To estimate the sample size required to address the primary research aim, an a priori sample size calculation was executed with G*Power software (version 3.1.9.6). The primary research question was analyzed with a mixed design ANOVA, with a two‐level between‐group factor (patients with FMS vs. healthy controls) and a three‐level within‐group factor (placebo learning mechanisms). Due to a lack of comparable studies, the required effect size for the primary research question was estimated to be moderate according to Cohen's “rule of thumb.”^32^ Entering a partial‐$\eta^{2}$ of 0.06 (*F*(v) = 0.25) in G*Power (1988), an alpha level of 0.05 and power of 0.80 yielded a required sample size of 156 (78 per group) complete cases. Furthermore, to compare the outcomes from respondents directly, both groups were matched based on their sex (male/female) and age category (18–40 years, 41–60 years, and 61 and above).

Respondents who filled in the entire questionnaire could win €25, with a raffle that was held at the end of the experiment. A total of 194 completed questionnaires were required for the entire project. Out of these, 10 respondents were given the financial award, which came down to a winning chance of 5%.

### Procedure

The questionnaire was conducted through Qualtrics software (April 2022 version; Qualtrics, Provo, UT, USA) and could be answered on a mobile phone, tablet, or computer. Respondents were invited to the advertisement to click a link to reach the questionnaire. At the beginning of the questionnaire, respondents were informed that they would fill in a questionnaire about opinions and attitudes toward placebo and nocebo effects. They were then asked to sign an online informed consent form. Following this, several screening questions had to be answered and if eligible, respondents could proceed to the main questionnaire.

The main survey consisted of six subsections, asking about: (1) sample demographics, symptoms related to fibromyalgia, and general attitudes toward medication and trust in physicians, (2) knowledge about placebo and nocebo effects, (3) perceived influence of placebo and nocebo contextual factors on outcome expectations, (4) acceptability of placebo‐based strategies and nocebo‐countering strategies, (5) perceived effectiveness of placebo‐based strategies and nocebo‐countering strategies, and (6) perceived influence of placebo and nocebo learning mechanisms (instructional, associative, and observational learning) on pain relief for three daily‐life treatment scenarios. In this sub‐study, the analyses were conducted with data from items about placebo effects in all but subsection 3, which assessed the influence of contextual factors specifically (see Supplemental Materials Item A, B, and C in Appendix S2). The order of the subsections itself was fixed, but the order of the items *within* the subsections, apart from the first subsection, was at random for every respondent. The entire survey took ~30 min to fill in. Respondents could pause the questionnaire at any moment and resume filling it in later (within 1 week). When all answers were submitted, respondents were asked to leave their e‐mail addresses if they wanted to participate in the raffle at the end of the experiment.

### Measures

#### Perceived influence of placebo learning mechanisms on pain relief

Assessing the respondents' perceived influence of placebo learning mechanisms on pain relief was done with three different scenarios, one for every learning mechanism. The scenario described someone experiencing headache for which they initially used over‐the‐counter analgesics and experienced pain relief. During a second headache episode occurring just a week after, the person would either: (1) visit a medical doctor who would recommend taking the analgesics (verbal suggestion), (2) again use it themselves (classical conditioning), or (3) observe a friend who benefits from the same analgesics (social observational learning). Right after taking the over‐the‐counter analgesics for the second time, the person noticed stronger pain relief. The respondents were asked to indicate the perceived influence of the learning manipulation on their assumed pain relief (ie, placebo effect) if they were the main subject of the scenario. For example: “To what degree do the instructions of the doctor determine the amount of pain relief if you were the person with headache in this story?”. The question was answered on a Numeric Rating Scale (NRS) scale ranging from 0 (not determining treatment outcome at all) to 10 (fully determining treatment outcome). The description of the scenarios and items can be found in the Supplemental Materials (Item C) in Appendix S2.

#### Acceptability and perceived effectiveness of placebo‐based strategies

Respondents were asked to rate how acceptable or effective they deemed the application of certain placebo‐based strategies to be. They were presented with five different types of strategies: (1) general placebo strategies, (2) closed‐label (ie deceptive) strategies, (3) open‐label strategies, (4) treatment‐enhancing strategies, and (5) dose‐extending strategies. The description of the items can be found in the Supplemental Materials (Item B) in Appendix S2. The amount of acceptability or perceived effectiveness for these five items was rated on an NRS scale ranging from 0 (totally not acceptable or totally not effective) to 10 (completely acceptable or completely effective). The items as well as their scoring were based upon previous placebo survey studies.^18^, ^20^, ^33^ Furthermore, to study the influence of medical history on symptoms related or unrelated to fibromyalgia, respondents were asked to rate the acceptability of placebo‐based strategies in different symptom categories (ie, acute pain, chronic pain, psychological symptoms, or insomnia).

#### Placebo knowledge

Respondents' knowledge about placebo effects was assessed with a 7‐item quiz based on a quiz created in a similar study by our research group.^33^ The original quiz contained 14 questions related to placebo knowledge. The questions could be answered as “correct” or “incorrect.” The shortened, 7‐item version of the placebo knowledge quiz was subsequently created with a dichotomous two‐parameter (2‐PL) model of the item response theory in R software environment,^34^ with the ltm package. We set out to select the seven items with the highest discrimination parameters and largest overall variance in difficulty to the previous 14‐item quiz. To do so, the two questions with the lowest and highest difficulty parameters were selected as the first and last question, respectively. To complete the set of seven items, the five remaining questions were selected by evaluating all possible subsets and subsequently selecting the subset with the highest model fit, effectively using the original quiz dataset as the training set. The placebo knowledge level of respondents was calculated with percentage scores based on the sum of all correct answers divided by the total amount of items.

#### Fibromyalgia diagnostic criteria

A Dutch version of the Fibromyalgia Survey Questionnaire (FSQ) was incorporated to describe the samples and to verify whether the self‐reported fibromyalgia diagnosis (or the lack of it) was accurately reported in both groups.^35^ The FSQ consists of two main sections: the symptom severity score (SSS) and the widespread pain index (WPI), and one final item about disease duration. The combination of the two main sections forms the Fibromyalgianess Scale (FS). The diagnosis is confirmed with the Fibromyalgia Survey Diagnostic Criteria, which consist of three facets: (1) Widespread Pain Index (WPI) ≥7/19 pain sites and Symptom Severity Score (SSS) ≥5/12, or WPI between 3–6/19 and SSS ≥9/12; (2) Symptoms have been present at a similar level for at least 3 months; (3) The patient does not have another disorder that would otherwise sufficiently explain the pain. The FSQ has good internal consistency (*α*_FS_ = 0.71) and good convergent and discriminant validity.^35^

### Statistical analysis

The data were analyzed with IBM SPSS statistics software (version 27). Descriptive statistics were applied to all measures; means and standard deviations for continuous outcomes, and frequencies and percentages for dichotomous outcomes. Statistical significance was considered at *p* < 0.05 for all main analyses, but adapted when conducting multiple comparisons with a Bonferroni correction.^36^ More specifically, the alpha level for the multiple comparisons was set to 0.008 in the primary analysis, and 0.005 in the secondary analyses. Effect sizes were shown as $\;\eta^{2}$ for one‐way ANOVAs, $\eta_{p}^{2}$ for mixed‐model AN(C)OVAs, Cohen's *d* for independent samples *t*‐tests, and (non‐standardized) regression coefficients for linear models.^37^

The primary analysis was set out to be conducted with a mixed‐model AN(C)OVA. The between‐subjects factor entered into the model was the two study groups (patients with FMS and healthy controls). The repeated within‐subjects factor was the different placebo learning mechanisms (verbal suggestions, classical conditioning, and social observational learning). The NRS scores of the perceived influence of placebo learning mechanisms on pain relief were the outcome of the primary analysis. Finally, as age or sex could be associated with differences in opinions about placebo effects,^38^, ^39^ they were entered as covariates. If both covariates were not significantly associated with the outcome, as was the case in a previous study,^20^ the analysis was run with a mixed‐model ANOVA instead of ANCOVA to optimize statistical power.

The secondary analyses concerned respondents' acceptability toward application of different placebo‐based strategies in clinical practice, or acceptability of these strategies applied in different clinical symptoms, or their perceived effectiveness. They were analyzed with separate mixed‐model AN(C)OVAs. Comparable to the primary analysis, the two study groups were entered as the between‐subjects factor. The repeated within‐subjects factors for the acceptability or perceived effectiveness models were the different placebo‐based strategies (general placebo strategies, closed‐label strategies, open‐label strategies, treatment‐enhancing strategies, and dose‐extending strategies). Whereas for the acceptability in the clinical symptoms model, the within‐subjects factor was the different symptoms (acute pain, chronic pain, insomnia, and psychological symptoms). The NRS scores of the acceptability scale, the acceptability in different symptoms scale, or the perceived effectiveness scale were the outcomes of the secondary analyses and, similarly to the primary analysis, age and sex were considered as covariates. Again, if both age and sex were not significantly associated with the amount of acceptability or perceived effectiveness, the analyses were instead run using mixed‐model ANOVAs.

The possible predictive role of respondents' perceived effectiveness of a placebo‐based strategy on their acceptability was exploratively analyzed with a multilevel linear mixed model (LMM). In this linear model, the relation between perceived effectiveness and acceptability was defined in the first level, whereas the role of a placebo‐based strategy in this relationship was defined in the second level. Integrating both levels resulted in a multilevel mixed model with three fixed factors: (1) perceived effectiveness, (2) placebo strategy, and (3) the interaction of perceived effectiveness × placebo strategy. It was further completed with three random factors: (1) a random intercept, (2) a random slope for the relationship of perceived effectiveness and acceptability, and (3) a random error term for each respondent. The full details of the multilevel LMM are presented in the Supplemental Materials Item D in Appendix S2. Differences in levels of placebo knowledge between groups were analyzed with independent samples *t*‐tests. Furthermore, two previous studies showed that self‐perceived placebo knowledge was not related to respondents' perceived acceptability or effectiveness; however, increased knowledge due to an educational intervention did increase both outcomes.^40^, ^41^ As the current survey examined similar constructs, the possible relationship of respondents' knowledge with the perceived acceptability or effectiveness toward the placebo‐based strategies was explored with the same mixed‐model ANCOVAs as the planned analyses, but now with knowledge entered as a covariate.

The assumptions for the independent samples *t*‐tests, AN(C)OVAs, and linear models were assessed by inspecting the distribution of the obtained variable scores with histograms and Q‐Q plots to check normality, Levene's test and Mauchly's test to check for the homogeneity of variances and sphericity of the repeated measures, residual plots to verify the presence of homoscedasticity, and non‐significant interaction between the independent variable and covariate to check the presence of homogeneity of regression. In case of a violation of the assumptions for a statistical test, suitable alternative testing was applied. In the case of non‐normality or the presence of heteroscedasticity, generalized linear models were conducted. When there was no homogeneity of the variances between groups, LMMs with a Satterthwaite approximation were conducted.^42^ Finally, a lack of sphericity in the data was corrected with Greenhouse–Geisser corrections.

## RESULTS

### Sample characteristics

For the larger study with all sub‐parts, a total of 444 responses were collected of which 185 had completed the entire survey and eventually 79 patients with FMS and 79 healthy controls were matched based on sex (male/female) and age category (18–40, 41–60, and 61 and above). After matching, both groups had a 4 (5%) to 75 (95%) male‐to‐female ratio, and 24 respondents (30%) belonging to the 18–40 age category, 40 respondents (51%) belonging to the 41–60 age category, and 15 respondents (19%) belonging to the 61 and above age category. Both groups had a median age that did not statistically differ from each other (patients: 49, healthy controls: 53, Mann Whitney *U*; 3250.00, *p* = 0.652). Age was not related to any of the outcomes as a covariate in the original ANCOVAs for the primary and secondary analyses. No robust conclusions could be made about the influence of sex as only a small amount of males could be matched in the analyses. It was therefore decided that the mixed‐model ANOVAs were conducted without age and sex as covariates. The role of sex was post hoc studied with an exploratory analysis of all healthy females and males (ie, when unmatched respondents were also included; see “Sex”). The FSQ revealed that a total of 66 (84%) patients with FMS met the diagnostic criteria for fibromyalgia syndrome compared with 2 (3%) healthy controls. To study the impact of this more stringent symptom questionnaire in fibromyalgia, patients meeting the FSQ diagnostic criteria were compared with healthy controls not meeting these criteria for all previously analyzed study outcomes in a post hoc exploratory analysis (see “FSQ diagnostic criteria”). For a more elaborate overview of the demographic data, see Table S1.

### Perceived influence of placebo learning mechanisms on pain relief

Upon checking the assumptions for the primary analysis, it was discovered that the obtained variable scores for the learning mechanisms were not perfectly normally distributed. However, the ANOVA was still preferred over non‐parametric testing, since ANOVAs are fairly robust to deviations from sample normality when an adequate sample size is obtained.^43^ The other assumptions for the ANOVA were all met. Patients with FMS and healthy controls did not differ, on average, in their perceived influence of the placebo learning mechanisms on pain relief, as was shown by a non‐significant main effect for groups (*F*(1, 156) = 1.535, *p* = 0.217, $\eta_{p}^{2}$ = 0.01). The main effect for the within‐subjects factor ‘learning mechanisms’ was statistically significant (*F*(1.92, 299.35) = 4.998, *p* = 0.008, $\eta_{p}^{2}$ = 0.03) indicating that across groups, there was a difference in the perceived influence of placebo learning mechanisms on pain relief. However, this difference did not vary per group as the interaction of group × learning mechanism was not significant (*F*(1.92, 299.35) = 0.574, *p* = 0.557, $\eta_{p}^{2}$ = 0.004). Post hoc comparisons between placebo learning mechanisms revealed that across groups, conditioning was perceived to be significantly more influential to pain relief than observational learning (mean difference [MD] = 0.449, standard error [SE] = 0.128, *p* < 0.001). No other differences between the placebo learning mechanisms were discovered (*p* ≥ 0.008). The descriptives and primary analyses are shown in Table 1, whereas the pairwise comparison between learning mechanisms is shown in Table S2.

**TABLE 1:** Descriptive and inferential statistics of the primary and secondary analyses.

Groups	Patients with FMS	Healthy controls	Main effect between groups
Outcomes	Mean (SD)	Mean (SD)	
Perceived influence of learning on pain relief (0–10 NRS)			*F*(1, 156) = 1.535, *p* = 0.217, $\eta_{p}^{2}$ = 0.01
Verbal suggestion	5.47 (2.36)	5.76 (2.54)	
Classical conditioning	5.49 (2.25)	6.09 (2.54)	
Observational learning	5.14 (2.23)	5.54 (2.57)	
Main effect across groups	*F*(1.92, 299.35) = 4.998, *p* = 0.008**, $\eta_{p}^{2}$ = 0.03		
Acceptability of Strategies (0–10 NRS)			*F*(1, 156) = 16.643, *p* < 0.001**
General	5.61 (2.71)	6.68 (2.11)	
Closed‐label	3.96 (3.01)	5.30 (2.53)	
Open‐label	5.54 (2.46)	6.62 (2.23)	
Treatment‐enhancing	6.37 (2.67)	7.89 (1.97)	
Dose‐extending	6.58 (2.40)	7.29 (2.10)	Interaction effect: *F*(4, 156) = 2.954, *p* = 0.020
Main effect across groups	*F*(4, 156) = 43.513, *p* < 0.001**		
Acceptability in Symptoms (0–10 NRS)			*F*(1, 156) = 10.419, *p* = 0.002**
Acute pain	4.33 (2.74)	5.62 (2.69)	
Chronic pain	4.86 (3.11)	5.99 (2.63)	
Psychological	5.25 (2.91)	6.28 (2.64)	
Insomnia	5.29 (3.02)	6.86 (2.22)	
Main effect across groups	*F*(3, 156) = 17.298, *p* < 0.001**		
Perceived Effectiveness of Strategies (0–10 NRS)			*F*(1, 156) = 12.132, *p* < 0.001**
General	5.23 (2.12)	6.04 (1.75)	
Closed‐label	5.76 (2.36)	6.85 (1.88)	
Open‐label	3.52 (2.34)	4.22 (2.13)	
Treatment‐enhancing	5.68 (2.43)	6.92 (1.66)	
Dose‐extending	5.03 (2.28)	5.92 (2.08)	
Main effect across groups	*F*(4, 156) = 46.029, *p* < 0.001**		
Placebo knowledge (% correct answers)	0.75 (0.20)	0.78 (0.17)	*t* = −1.237, *p* = 0.218, *d* = −0.20

*Note*: Results from the primary and secondary analyses. The difference in groups for an outcome (eg, perceived influence of verbal suggestions between groups) was compared when the interaction effect for that model showed significance. However, if merely the main effect was significant, the comparisons were drawn either across outcomes (“between‐group effect”) or across groups (“within‐group effect”). ** *p* < 0.001

Abbreviations: NRS, Numeric Rating Scale; SD, standard deviation.

### Acceptability of placebo‐based strategies and the application of placebo effects in clinical symptoms

The assumptions for the secondary analyses were not all completely met. The amount of variance for the acceptability of general placebo strategies, closed‐label strategies, treatment‐enhancing strategies, and the acceptability in chronic pain and insomnia was not homogeneous (Levene's test: *p* < 0.05). Therefore, instead of ANOVAs, the analyses were conducted with LMMs following the Satterthwaite approximation. There was a statistically significant difference in overall placebo acceptability between patients with FMS and healthy controls, according to the main effect for the group (*F*(1, 156) = 16.64, *p* < 0.001). Patients considered placebo‐based strategies less acceptable compared with healthy controls. The main within‐subject effect of the different placebo‐based strategies on acceptability was also significant (*F*(4, 156) = 43.513, *p* < 0.001), which implied that the levels of acceptability for different placebo‐based strategies significantly varied. This did vary per group, as the interaction term of group × strategy was also statistically significant (*F*(4, 156) = 2.954, *p* = 0.02). Pairwise comparisons of groups per strategy revealed that patients with FMS considered closed‐label strategies and treatment‐enhancing strategies to be significantly less acceptable than healthy controls (closed‐label: MD = −1.342, SE = 0.442, *p* = 0.003; treatment‐enhancing: MD = −1.519, SE = 0.373, *p* < 0.001), whereas for the other strategies, groups did not significantly differ in acceptability. Furthermore, within patients, the use of closed‐label strategies was significantly less acceptable than any other strategy (all *p* < 0.001), whereas general strategies were considered equally acceptable as open‐label strategies (*p* = 0.854), but less acceptable than treatment‐enhancing (*p* < 0.001) or dose‐extending strategies (*p* < 0.001). Patients considered the latter two strategies equally acceptable (*p* = 0.230). In healthy controls, closed‐label strategies were also significantly less acceptable than the remaining strategies (all *p* < 0.005), and open‐label strategies were as acceptable as general strategies (*p* = 0.854). However, healthy controls considered treatment‐enhancing strategies significantly more acceptable than general placebo strategies (*p* < 0.001) and open‐label strategies (*p* < 0.001), whereas dose‐extending strategies were not considered more acceptable than general strategies (*p* = 0.009) and open‐label strategies (*p* = 0.048). For all results, see Tables S3 and S4.

The acceptability toward placebo‐based strategies applied in various symptom categories also differed between patients with FMS and healthy controls (*F*(1, 156) = 10.42, *p* = 0.002). Patients considered the application of placebo‐based strategies less acceptable than healthy controls across symptoms. The amount of acceptability per symptom category also differed across groups (*F*(4, 156) = 46.029, *p* < 0.001), but there was no significant interaction of group × symptom category (*F*(3, 156) = 1664, *p* = 0.177). Post hoc comparisons for symptom categories across groups showed that placebo‐based strategies were seen as more acceptable in chronic pain, psychological symptoms, or insomnia than in acute pain, and more acceptable in insomnia as compared with chronic pain, with the other symptoms not significantly differing from each other (see Table S5).

### Perceived effectiveness of placebo‐based strategies

Similar to the analyses for acceptability, the variance for the perceived effectiveness of general placebo strategies, closed‐label strategies, and treatment‐enhancing strategies was not homogeneous (Levene's test: *p* < 0.05). Therefore, instead of ANOVAs, the analyses were conducted with LMMs following the Satterthwaite approximation, similar to the acceptability analyses. Patients with FMS and healthy controls differed significantly in the overall perceived effectiveness of placebo‐based strategies, which was shown by the between‐subjects effect of the ANOVA (*F*(1, 156) = 12.132, *p* < 0.001). Patients perceived the placebo‐based strategies to be less effective than healthy controls. The main within‐subjects effects showed that across groups, the perceived effectiveness for certain placebo‐based strategies differed (*F*(4, 156) = 46.029, *p* < 0.001), yet it did not vary per group, as the interaction effect of groups × strategy was not significant (*F*(4, 156) = 1.363, *p* = 0.249). The post hoc comparisons for the different placebo‐based strategies across groups revealed that open‐label strategies were perceived as significantly less effective than the other placebo‐based strategies. For the remaining comparisons, closed‐label strategies, or treatment‐enhancing strategies were considered to be significantly more effective than general placebo strategies, or dose‐extending strategies (see Table S6).

### Predictive role of perceived effectiveness on acceptability

The multilevel LMM showed that higher perceived effectiveness across placebo‐based strategies was significantly related to higher acceptability of these strategies (*F*(1, 400.99) = 316.584, *p* < 0.0001, *b* = 0.555, SE = 0.067). In addition, the interaction term strategy × perceived effectiveness was significant (*F*(4, 607.62) = 5.075, *p* < 0.001), which indicated that the strength of the relationship between perceived effectiveness and acceptability varied between placebo‐based strategies. More specifically, the magnitude of the relationship between perceived effectiveness and acceptability for open‐label strategies and dose‐extending strategies was comparable, whereas this relationship was stronger for general placebo strategies, closed‐label strategies, and treatment‐enhancing strategies (see Table S7).

### Placebo knowledge

The cumulative scores of respondents regarding knowledge of placebo effects were overall high (*M* = 0.77, SD = 0.18), see also Table S8. An independent samples *t*‐test showed that both groups did not significantly differ in knowledge scores (*t* = −1.24, *p* = 0.218, *d* = −0.20). Since the homogeneity of variance assumption was not met for the ANOVAs of the acceptability, acceptability in different symptoms, and perceived effectiveness analyses, the role of knowledge as a covariate was studied by imputing it in the subsequent LMMs. The results showed that across groups, higher placebo knowledge was significantly associated with higher acceptability (*F*(1, 155) = 4.801, *p* = 0.03) and perceived effectiveness (*F*(1, 155) = 11.735, *p* < 0.001) of placebo‐based strategies. The associations did not vary between groups, placebo‐based strategies, or the interaction of both predictors, as the interaction terms for these predictors with knowledge for both outcomes were not statistically significant (acceptability_groups*knowledge_: *F*(5, 144) = 0.537, *p* = 0.748, acceptability_strategy*knowledge_: *F*(28, 144) = 0.952, *p* = 0.540, acceptability_groups*strategy*knowledge_: *F*(20, 144) = 0.902, *p* = 0.585, perceived efficacy_groups*knowledge_: *F*(5, 144) = 0.439, *p* = 0.821, perceived efficacy_strategy*knowledge_: *F*(28, 144) = 0.890, *p* = 0.627, perceived efficacy_groups*strategy*knowledge_: *F*(20, 144 = 0.795, *p* = 0.495)) (see also Tables S9 and S10).

### Sex

**FIGURE 1:**
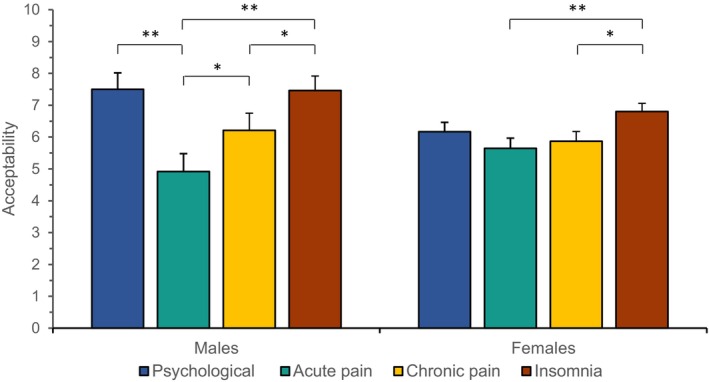
Acceptability of placebo‐based strategies per symptom category by healthy males and females. The bar chart shows how acceptable the healthy males or females considered the application of placebo‐based strategies for different symptom categories. The bars represent the average acceptability, whereas error bars depict the standard error of the mean. *Statistical significance at *p* < 0.005, **Statistical significance at *p* < 0.001.

The influence of sex was explored in post hoc analyses since males and females seem to respond differently to placebo effects,^38^, ^44^ although their opinions were previously not significantly different.^20^ Since few males with fibromyalgia filled out the survey, the differences between all healthy females (*N* = 75) and males (*N* = 24), including unmatched responses (total *N* = 99), was alternatively studied to compare sex. The analyses were conducted with ANOVAs, with sex as between‐subject factor and the different conditions of either outcome (eg, acceptability of open‐label placebo use) as the within‐subject factor. All assumptions were met. There were no differences in sex regarding the perceived influence of placebo‐based learning mechanisms on pain relief, the acceptability of placebo‐based strategies, and the perceived effectiveness of these strategies (all *p* ≥ 0.472, see also Tables S11–S13 and S16). There was a significant yet small interaction effect of sex with symptom categories regarding acceptability (*F*(2.77, 265.72) = 5.72, *p* = 0.001, $\eta_{p}^{2}$ = 0.06), indicating that the amount of acceptability when using placebo‐based strategies for different symptoms varied more in males compared with females. Post hoc comparisons showed that males considered the application of placebo‐based strategies in psychological symptoms more acceptable than females (MD = 1.327, SE = 0.595, *p* = 0.028). Males deemed the use of strategies in psychological symptoms and insomnia equally acceptable, but in acute pain, when compared with both these symptom categories, significantly less acceptable. Using placebo‐based strategies in chronic pain was considered significantly more acceptable than in acute pain, but significantly less acceptable than in insomnia, and borderline less acceptable than in psychological symptoms (*p* = 0.008). Females, however, indicated that the use of strategies was more acceptable in insomnia compared with either acute pain, or chronic pain, and borderline more acceptable compared with psychological symptoms (*p* = 0.008; see Figure 1 and Tables S14 and S15).

### FSQ diagnostic criteria

**FIGURE 2:**
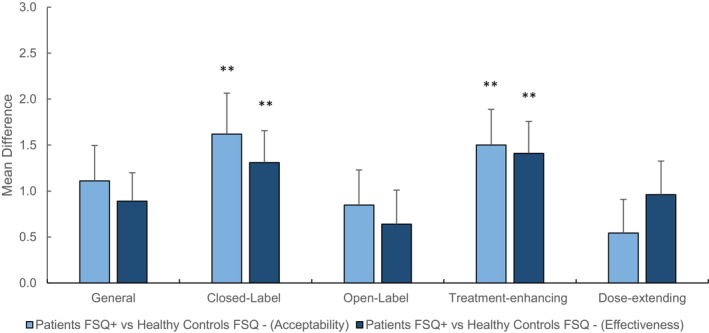
The mean difference in acceptability or perceived effectiveness of placebo‐based strategies between patients who met the FSQ criteria and healthy controls that did not meet the FSQ criteria. The bar graph shows the average difference scores between the two groups per strategy and error bars depict the standard error of the mean. The light blue bars on the left side show the mean differences for the acceptability ratings, whereas the dark blue bars on the right side depict the perceived effectiveness ratings. FSQ, Fibromyalgia Symptom Questionnaire. **Statistical significance at *p* < 0.001.

The difference in opinions between patients that met the FSQ criteria (*N* = 64) and healthy controls that did not (*N* = 77) was exploratively analyzed. To conduct this analysis, these subgroups were isolated and entered as the between‐subjects factor in either the ANOVAs or the LMMs of the primary and secondary outcomes (influence of placebo learning mechanisms, acceptability of placebo‐based strategies, acceptability for different symptoms, perceived effectiveness of placebo‐based strategies, and placebo knowledge) (see also Tables S17–S23). The results were similar to the primary and secondary analyses, apart from a significant interaction effect of groups × strategy for the perceived effectiveness outcome (See Figure 2 and Tables S22 and S23).

## DISCUSSION

This cross‐sectional survey study aimed to clarify the potential role of medical history (eg, patients vs. healthy individuals), moral standpoints (eg, acceptability), beliefs (eg, perceived effectiveness), and knowledge about implementation of placebo effects. Although patients and healthy controls did not differ in their perceived influence of the placebo learning mechanisms (instructional learning, conditioning, and observational learning), conditioning was overall deemed more effective in bringing about placebo effects in pain than observational learning. Also, although both groups showed relatively modest acceptability and perceived effectiveness of treatment strategies using placebo effects, healthy controls considered their application in clinical practice more acceptable in closed‐label strategies and treatment‐enhancing strategies, and more effective across all strategies than patients. Within both groups, respondents rated closed‐label strategies as least acceptable, and open‐label strategies as acceptable as general placebo strategies. Patients considered treatment‐enhancing strategies and dose‐extending strategies equally most acceptable, whereas healthy controls considered treatment‐enhancing strategies to be most acceptable, even more so than dose‐extending strategies. Insomnia was deemed the most acceptable symptom for clinical application of placebo‐based strategies and acute pain was the least acceptable. In terms of placebo effectiveness, respondents perceived closed‐label strategies, and treatment‐enhancing strategies to be the most effective, whereas open‐label strategies were assumed the least effective. Knowledge levels about placebo effects did not differ between groups, yet an exploratory analysis revealed that increased knowledge across groups was related to higher acceptability and perceived effectiveness of respondents toward the placebo‐based strategies. The degree of perceived effectiveness was also positively related to the acceptability of strategies.

The study showed for the first time that medical history differentiates how recipients judge acceptability and perceived effectiveness of clinical application of some placebo‐based strategies. Patients with fibromyalgia expressed significantly lower acceptability levels toward closed‐label and treatment‐enhancing strategies compared with healthy controls, whereas for the other strategies, the differences were not significant at *p* < 0.005. Patients are possibly more reserved toward implementation of placebo‐based applications when they are not fully informed about a treatment (ie closed‐label), or when they are merely added as “inert” treatment modalities to an existing treatment (ie, treatment‐enhancing) that has been proven effective. Withholding them from autonomy in the decision process for a certain treatment or suggesting them to add a so‐called “inert” medical treatment to their existing treatment could lead to strong feelings of invalidation.^28^ Also, they could be skeptical about any sham treatment's effectiveness because of their previous experience with ineffective medical approaches,^45^ which, in turn, could lead to lower acceptance rates. This was substantiated by the discovered relationship between acceptability and perceived effectiveness in this study and previous related surveys.^18^ Interestingly, when patients meeting the FSQ diagnostic criteria were compared with healthy controls not meeting these, the relationship between perceived effectiveness and acceptability was further highlighted as both groups showed significant differences for the same strategies (eg, closed‐label, and treatment‐enhancing strategies). All in all, these reasonings could have further shaped the acceptability of patients toward implementation of placebo‐based strategies, in comparison to healthy controls, and stressed the importance of highlighting both opinions.

Apart from the role of medical history, various important findings from this study were concluded from concepts (eg, acceptability, perceived effectiveness, and knowledge) that were also previously studied.^18^, ^19^, ^20^, ^21^, ^24^, ^33^, ^40^, ^46^ For instance, disclosing to recipients about the nature of a placebo‐based strategy, that is open‐label, is more acceptable than concealment of a strategy, that is, closed‐label,^20^, ^21^, ^29^ and this is likely because individuals value their right to be informed.^7^ Furthermore, the amount of acceptability relates partially to how effective individuals assume placebo‐based strategies to be,^21^, ^29^, ^40^ an ethical principle known as consequentialism.^47^ Another important finding is that strategies are deemed more acceptable when they are used to enhance or substitute an existing treatment modality instead of when used as an isolated intervention.^19^, ^20^ This conception could stem from the idea that recipients might feel more acceptant of a placebo‐based strategy that does not withhold them from receiving a well‐established medical treatment,^19^ which is known as the principle of clinical equipoise.^48^ Interestingly, these ethical principles seem to have a varying influence on someone's opinion depending on the conditions of a strategy. For instance, although closed‐label strategies were considered one of the most effective placebo‐based strategies, their acceptability was actually the lowest, possibly because concealment negatively impacts a patients' right to be informed. In addition, individuals considered substituting placebo‐based strategies (ie, dose‐extending) more acceptable than closed‐label strategies despite their lower perceived effectiveness, which suggests that clinical equipoise prevails over consequentialism. Ultimately, these findings indicate that someone's evaluation of how acceptable or effective placebo‐based strategies are, rely on their knowledge about placebo effects. Similar to previous studies, the current results indeed confirmed this as a positive relation between knowledge, perceived effectiveness, and acceptability of placebo‐based strategies was discovered.^41^ However, the lower acceptance rates of patients compared with healthy controls begs the question whether improving patients' knowledge about placebo‐based strategies, for instance, concerning open‐label strategies can increase acceptance rates in clinical practice.

### Clinical implications

Despite the overwhelming evidence for the effectiveness of placebo‐based strategies in fundamental and clinical research,^49^, ^50^, ^51^ implementation of these strategies in clinical practice is still at its infancy.^2^ The views of respondents about placebo effects and concomitant placebo‐based strategies discovered in this study aid in selecting methods that are not only effective, but also considered permittable by the ones receiving them. Ultimately, the results contribute to a patient‐centered implementation of placebo‐based strategies in clinical practice, such as shared decision‐making (SDM), and this could increase patients' risk estimation and adherence to treatments.^52^, ^53^, ^54^ Since our results also emphasized the relevance of knowledge about placebo‐based strategies in foreseeing their effectiveness and, in turn, being acceptable toward receiving them, improvement of knowledge about placebo effects by means of educational interventions could crucially aid clinical implementation.^41^ The use of open‐label placebo strategies, in which a physician actively informs a patient about the placebo admission, is one example that could therefore be adopted in clinical practice. Another clinically important finding is the higher ratings in perceived effectiveness of both treatment‐enhancing and dose‐extending strategies compared with the ratings of the remaining placebo‐based strategies by both patients and healthy people. As both groups seem similarly convinced about the optimization of treatment effects with add‐on or substituting strategies, making use of placebo effects through these strategies in clinical practice could be further explored.^8^ For example, dose‐extending strategies seem to be an interesting treatment option for the tapering of long‐term medication use in practice, although the deceptive element still hampers practical implementation. Interestingly, in a recently published protocol the use of dose‐extending placebos will therefore be combined with an open‐label placebo to avoid the deception.^55^ If the combination of these strategies proves to be successful, its adoption in clinical practice is a valid way of lowering long‐term medicine use by means of placebo‐based strategies.

### Limitations

There are several limitations that are noteworthy to mention for their influence in this study. First, the cross‐over design of the study limits the extent of the conclusions that can be drawn from some of the current findings. For instance, it is impossible to rule out that the positive linear relationship between knowledge, perceived effectiveness, and acceptability of the strategies is not in reversed order (eg, greater acceptability leads to more perceived effectiveness instead of the other way around). However, from a theoretical perspective, discovering such predictive relationships in reversed order would be very unlikely.^18^, ^41^ Second, the recruitment of patients with fibromyalgia, which was performed online because of the COVID‐19 pandemic, could have induced selection bias. If patients were skeptical of placebo effects, they might have avoided filling in the survey, leading to possibly positively skewed results favoring acceptability of strategies. Compared with a previous study, the current acceptability ratings for placebo‐based strategies by patients were indeed higher, although, relatively speaking, the results were comparable.^20^ Third, the current opinions from patients with fibromyalgia are not generalizable to other chronic pain syndromes, which could limit the extent of the insights from this survey. However, a previous survey did not find differences in acceptability ratings based on the chronic pain diagnosis and one could thus argue that the current opinions do give some indication of the views of other chronic pain patients.^20^ Fourth, despite being used by multiple other survey studies,^18^, ^20^, ^21^, ^40^ the psychometric characteristics of the items about the acceptability and perceived effectiveness of the placebo‐based strategies has never been completely assessed, although the test–retest reliability in physicians was strong.^18^ Therefore, the results from this survey study, and the previous line of work, have to be carefully compared, although all of these findings seem largely in line with one another. Fifth, the low amount of males in each group made it practically impossible to draw conclusions about the role of sex due to the low amount of statistical power. As such, a second‐best exploratory analysis was conducted to gain more insight on this topic, but a possible interaction by the study groups and sex could resultantly have been missed. Sixth, the duration of the FMS disease symptoms, which could influence a patients' opinion due to the prolonged experience with clinical practice, was not collected in the survey. Yet, most patients with FMS have a substantial duration in symptoms as, on average, the diagnosis is made 2 years after the on‐set of symptoms.^56^ Seventh, the average scores obtained by respondents on the placebo knowledge quiz was high and this lowered the variability of the results (ie, created ceiling effects). Similar results were found in the survey that this knowledge questionnaire was based upon, and efforts to increase the range of difficulty with IRT modeling did not have the desired effect of increasing variability.^33^ Regardless of these shortcomings, the carefully matched design and sufficient sample size in this study were two predominant strengths that made a thorough comparison between patients and healthy controls possible.

## CONCLUSION

This survey showed that a persons' medical history relates to acceptability and perceived effectiveness toward clinical implementation of placebo‐based strategies. Healthy controls deemed closed‐label strategies and treatment‐enhancing strategies more acceptable than patients, whereas healthy controls deemed the placebo‐based strategies in general more effective than patients. Furthermore, the results emphasized that strategies with more transparency, or higher perceived effectiveness, or in adjunction to existing medical treatments were related to greater acceptability of their application. Knowledge about placebo effects was also positively related to both acceptability and perceived effectiveness. In summary, the findings suggest that someone's medical history, right to be informed, expectations about effectiveness, and specific placebo knowledge may influence personal judgments about the implementation of strategies aimed at inducing placebo effects in clinical practice. Providing adequate educational interventions seems essential to implement placebo effects further into clinical practice.

## AUTHOR CONTRIBUTIONS

All authors have contributed substantially to the manuscript.

## FUNDING INFORMATION

This research was funded by the Dutch Arthritis Society (grant number BP18‐1‐501), an NWO VICI grant (grant number 45316004), and an NWO Stevin grant (grant number Stevin2019.1) of the Netherlands Organization for Scientific Research, granted to A. Evers.

## CONFLICT OF INTEREST STATEMENT

The authors have no conflicts of interest to declare.

## PATIENT CONSENT

Respondents filled out an online informed consent form through Qualtrics software.

## Supporting information


Appendix S1

Appendix S2

## Data Availability

All experimental data (with the exception of personal identifiable data) will be made publicly available through the online university data repository (DataverseNL).
