# Supplementary material for: Do patients with fibromyalgia syndrome and healthy people differ in their opinions on placebo effects in routine medical care?
Source: Pain Pract. 2025 Jan 27;25(2):e70000. doi: 10.1111/papr.70000 (PMC11771638; doi:10.1111/papr.70000)
Supplement: Supplementary file 1 — Appendix S1 [file PAPR-25-0-s002.docx]

**Do patients with fibromyalgia syndrome and healthy people differ in their opinions on placebo effects in routine medical care?**

**Supplemental Materials**

**Placebo-related items from the study survey that have been used in the current study**

**Item A. Placebo knowledge quiz**

*Placebo and nocebo effects are psychological mechanisms that have a positive or negative influence on the outcome of a treatment and/or symptoms that one experiences. The next quiz aims to discover the amount of knowledge you have of both effects. De quiz consists of two parts with similar statements. One part is about placebo effects and the other about nocebo effects.*

*Please indicate whether the statements below about placebo effects are correct or incorrect.*

|  | | Correct | Incorrect | *Answer* |
| --- | --- | --- | --- | --- |
| 1. 1. | 1. Emotions and cognitions (e.g., trust) about the physician and medical treatments contribute to placebo effects. |  |  | *Correct* |
| 1. 2. | Placebo effects do not work when a person knows he or she is taking a placebo. |  |  | *Incorrect* |
| 3. | Placebo effects only occur in alternative medicine (such as acupuncture or herbal treatment). |  |  | *Incorrect* |
| 4. | The packaging of a placebo (e.g., color of the pill) can influence its effects. |  |  | *Correct* |
| 5. | A placebo or inert substance (e.g. sugar pills) can reduce symptoms such as pain. |  |  | *Correct* |
| 6. | Placebo effects only occur in scientific research. |  |  | *Incorrect* |
| 7. | Placebo effects only occur in psychological complaints (such as stress). |  |  | *Incorrect* |

**Item B.** **Acceptability and perceived effectiveness of placebo-evoking strategies**

*The next questions are about placebo effects. Placebo effects are* ***positive*** *treatment effects that are not the result of the active treatment components but can, for example, be the result of* ***positive*** *patient views of a treatment. Placebo effects can be created by instructions about a treatment, yet can also be acquired (for example through previous experiences with treatments).*

*In order to give rise to placebo effects, doctors can make use of placebo treatments. A placebo treatment is essentially a treatment without medically-active components, through which the doctor tries to relief a patients’ symptoms. A well-known example in every day life is the “kiss against the pain” that is often provided by a mother to her child. Placebo treatments can also temporarily substitute or enhance an active treatment.*

The following questions aim to discover how **acceptable** you deem such treatments.

How acceptable do you consider placebo treatments?

| Completely unacceptable Neutral Completely acceptable | | | | | | | | | | |
| --- | --- | --- | --- | --- | --- | --- | --- | --- | --- | --- |
|  | | | | | | | | | | |
| 0 | 1 | 2 | 3 | 4 | 5 | 6 | 7 | 8 | 9 | 10 |

How acceptable would you consider a physician using a placebo treatment **without** telling the patient?

| Completely unacceptable Neutral Completely acceptable | | | | | | | | | | |
| --- | --- | --- | --- | --- | --- | --- | --- | --- | --- | --- |
|  | | | | | | | | | | |
| 0 | 1 | 2 | 3 | 4 | 5 | 6 | 7 | 8 | 9 | 10 |

How acceptable would you consider a physician using a placebo treatment **when telling** the patient?

| Completely unacceptable Neutral Completely acceptable | | | | | | | | | | |
| --- | --- | --- | --- | --- | --- | --- | --- | --- | --- | --- |
|  | | | | | | | | | | |
| 0 | 1 | 2 | 3 | 4 | 5 | 6 | 7 | 8 | 9 | 10 |

How acceptable would you consider a physician adding-on a placebo treatment to a regular pain treatment in order to enhance the effectiveness thereof for the patient?

| Completely unacceptable Neutral Completely acceptable | | | | | | | | | | |
| --- | --- | --- | --- | --- | --- | --- | --- | --- | --- | --- |
|  | | | | | | | | | | |
| 0 | 1 | 2 | 3 | 4 | 5 | 6 | 7 | 8 | 9 | 10 |

How acceptable would you consider a physician using a placebo treatment that has been proven to mimic the effects of a drug, in order to see whether this may **substitute** part of the medication of a patient?

| Completely unacceptable Neutral Completely acceptable | | | | | | | | | | |
| --- | --- | --- | --- | --- | --- | --- | --- | --- | --- | --- |
|  | | | | | | | | | | |
| 0 | 1 | 2 | 3 | 4 | 5 | 6 | 7 | 8 | 9 | 10 |

*The previous questions were about how acceptable you consider certain placebo treatments. We would also like to ask you how acceptable you deem such placebo treatments for the treatment of different symptoms (e.g., pain or anxiety).*

How acceptable do you consider placebo treatments for the treatment of:

…psychological symptoms (anxiety, depression)?

| Completely unacceptable Neutral Completely acceptable | | | | | | | | | | |
| --- | --- | --- | --- | --- | --- | --- | --- | --- | --- | --- |
|  | | | | | | | | | | |
| 0 | 1 | 2 | 3 | 4 | 5 | 6 | 7 | 8 | 9 | 10 |

…acute pain symptoms (headache, abdominal pain, strain)?

| Completely unacceptable Neutral Completely acceptable | | | | | | | | | | |
| --- | --- | --- | --- | --- | --- | --- | --- | --- | --- | --- |
|  | | | | | | | | | | |
| 0 | 1 | 2 | 3 | 4 | 5 | 6 | 7 | 8 | 9 | 10 |

…chronic pain symptoms (back pain, muscle pain, nerve pain)?

| Completely unacceptable Neutral Completely acceptable | | | | | | | | | | |
| --- | --- | --- | --- | --- | --- | --- | --- | --- | --- | --- |
|  | | | | | | | | | | |
| 0 | 1 | 2 | 3 | 4 | 5 | 6 | 7 | 8 | 9 | 10 |

…insomnia?

| Completely unacceptable Neutral Completely acceptable | | | | | | | | | | |
| --- | --- | --- | --- | --- | --- | --- | --- | --- | --- | --- |
|  | | | | | | | | | | |
| 0 | 1 | 2 | 3 | 4 | 5 | 6 | 7 | 8 | 9 | 10 |

*The previous questions were about how acceptable you deemed placebo treatments. In addition, we would like to know how* ***effective*** *you think that such placebo treatments are?*

How effective do you consider placebo treatments in general?

| Totally ineffective Neutral Very effective | | | | | | | | | | |
| --- | --- | --- | --- | --- | --- | --- | --- | --- | --- | --- |
|  | | | | | | | | | | |
| 0 | 1 | 2 | 3 | 4 | 5 | 6 | 7 | 8 | 9 | 10 |

How effective do you consider a placebo treatment in which a physician **does not tell** the patient that they receive a placebo treatment?

| Totally ineffective Neutral Very effective | | | | | | | | | | |
| --- | --- | --- | --- | --- | --- | --- | --- | --- | --- | --- |
|  | | | | | | | | | | |
| 0 | 1 | 2 | 3 | 4 | 5 | 6 | 7 | 8 | 9 | 10 |

How effective do you consider a placebo treatment in which a physician **tells** a patient that they receive a placebo treatment?

| Totally ineffective Neutral Very effective | | | | | | | | | | |
| --- | --- | --- | --- | --- | --- | --- | --- | --- | --- | --- |
|  | | | | | | | | | | |
| 0 | 1 | 2 | 3 | 4 | 5 | 6 | 7 | 8 | 9 | 10 |

How effective do you consider a placebo treatment that is used by a physician to **enhance** the effectiveness of a medical treatment?

| Totally ineffective Neutral Very effective | | | | | | | | | | |
| --- | --- | --- | --- | --- | --- | --- | --- | --- | --- | --- |
|  | | | | | | | | | | |
| 0 | 1 | 2 | 3 | 4 | 5 | 6 | 7 | 8 | 9 | 10 |

How effective do you consider a placebo treatment that is used to **substitute** part of a medical treatment?

| Totally ineffective Neutral Very effective | | | | | | | | | | |
| --- | --- | --- | --- | --- | --- | --- | --- | --- | --- | --- |
|  | | | | | | | | | | |
| 0 | 1 | 2 | 3 | 4 | 5 | 6 | 7 | 8 | 9 | 10 |

**Item C. Influence of learning mechanisms on placebo effects**

*The following scenarios describe different ways to evoke placebo effects by means of a simple case description. We would like you to indicate per case how much influence you believe that such placebo effects would have if you were the person in the story.*

Scenario 1.

*Imagine the following: A person suffers from a headache and decides to pick up some painkillers at the drugstore. He takes the painkillers and notices a relief of the headache after half an hour. The next week the person suffers from the same headache. This time he is a bit more concerned about the complaints and as such visits a doctor and asks him about the painkillers that he has taken earlier. The doctor responds: The painkiller that you used has a strong effectiveness and should be adequate for your headache. After this instruction, the person again takes the painkillers and this time notices relief of the headache complaints after just 5 minutes.*

To what degree do the instructions of the doctor determine the amount of **pain relief** if you were the person with headache in this story?

| Not at all Completely | | | | | | | | | | |
| --- | --- | --- | --- | --- | --- | --- | --- | --- | --- | --- |
|  | | | | | | | | | | |
| 0 | 1 | 2 | 3 | 4 | 5 | 6 | 7 | 8 | 9 | 10 |

*Scenario 2.*

*Imagine the following: A person suffers from a headache and decides to pick up some painkillers at the drugstore to relieve the pain. He takes the painkillers and notices a relief of the headache after half an hour. The next week the person suffers from the same headache and visits the drugstore again to buy painkillers. After taking the painkillers, he notices himself that the pain complaints diminish already after a few minutes.*

To what degree do the previous experiences determine the amount of pain relief if you were the person with headache in this story?

| Not at all Completely | | | | | | | | | | |
| --- | --- | --- | --- | --- | --- | --- | --- | --- | --- | --- |
|  | | | | | | | | | | |
| 0 | 1 | 2 | 3 | 4 | 5 | 6 | 7 | 8 | 9 | 10 |

*Scenario 3.*

*Imagine the following: A person suffers from a headache and decides to pick up some painkillers at the drugstore to relieve the pain. He takes the painkillers and notices a relief of the headache after half an hour. After a week he visits a friend who also suffers from a headache to take care of him. The friend is in bed and asks him for some painkillers from his nightstand. The person notices that these are the same painkillers as the ones that he bought for his headache. Half an hour later the friend is experiencing significantly less pain and tells the person that he always benefits from these painkillers. After two weeks, the person again suffers from a headache. Fortunately, he still has some of the painkillers. After ingesting them, the person notices pain relief after just 5 minutes.*

To what degree does the observation of pain relief from the friend determine the amount of pain if you were the person with headache in this story?

| Not at all Completely | | | | | | | | | | |
| --- | --- | --- | --- | --- | --- | --- | --- | --- | --- | --- |
|  | | | | | | | | | | |
| 0 | 1 | 2 | 3 | 4 | 5 | 6 | 7 | 8 | 9 | 10 |

**Item D. Exploratory analysis: predictive value of perceived effectiveness on acceptability per placebo-based strategy.**

*The possible predictive role of respondents’ perceived effectiveness of a placebo-based strategy on their acceptability was exploratively analyzed with a multilevel linear mixed model (LMM). In this linear model, perceived effectiveness was entered as a fixed factor in the first level, whereas placebo-based strategy was defined as a fixed factor in the second level. Additionally, a random intercept, random slopes for the linear relation between perceived effectiveness and acceptability, and a random error term for each respondent were estimated.*

Analysis method: multilevel linear mixed model.

Level 1.

*Acceptability*

*Perceived Effectiveness*

Regression formula where perceived effectiveness is a fixed factor:

$$Acc= \beta_{0j}+ \beta_{1j} \times Eff+ \varepsilon_{i}$$

*General strategies*

Level 2.

*Acceptability*

*Perceived Effectiveness*

*Dose-extending strategies*

*Treatment-enhancing*

*Open-label strategies*

*Closed-label strategies*

Regression formula where the regression coefficient and intercept from level 1 are defined by: 1) a fixed intercept (Y0), 2) the fixed effect of strategy (Y1), or a random intercept (u).

$$\beta_{0j}= \gamma_{00}+ \gamma_{01}\times Strategy+ \mu_{0}$$

$$\beta_{1j}= \gamma_{10}+ \gamma_{11}\times Strategy+ \mu_{1}$$

Inserting regression coefficients onto level 1. The formula below yields the entire mixed regression model.

$$Acc= \gamma_{00}+ \gamma_{01}\times Strategy+ \gamma_{10}\times Eff+ \gamma_{11}\times Strategy \times Eff+ \mu_{0}+ \mu_{1}\times Eff+ \varepsilon_{i}$$

**Supplemental results**

**Table 1. Demographics**

| **Groups** | | **Patients with FMS** | | | **Healthy Controls** | | |
| --- | --- | --- | --- | --- | --- | --- | --- |
|  | | Males | Females | Total | Males | Females | Total |
| Age category | 18-40 | 0 (0%) | 24 (30%) | 24 (30%) | 0 (0%) | 24 (30%) | 24 (30%) |
|  | 41-60 | 1 (1%) | 39 (50%) | 40 (51%) | 1 (1%) | 39 (50%) | 40 (51%) |
|  | 61 + | 3 (4%) | 12 (15%) | 15 (19%) | 3 (4%) | 12 (15%) | 15 (19%) |
| Median Age (IQR) | | 64 (55;73) | 48 (28;68) | 49 (27;61) | 61.5 (47.8;75.2) | 52 (24;80) | 53 (26;70) |
| Sex | | 4 (5%) | 75 (95%) | 79 (100%) | 4 (2.5%) | 75 (95%) | 79 (100%) |
| Meeting FSQ diagnostic criteria | | 2 (3%) | 64 (81%) | 66 (84%) | 0 (0%) | 2 (3%) | 2 (3%) |

Demographic overview of the matched study samples. Respondents were matched by age and sex. The number of male or female patients per age category was matched to the same number of healthy controls. FSQ = Fibromyalgia Survey Questionnaire.

**Table 2. Difference in perceived influence of placebo learning mechanisms on pain relief *across* groups**

| **Placebo learning mechanisms** | **Pairwise comparisons** | |
| --- | --- | --- |
|  | Mean Difference (SE) | Significance level |
| Verbal Suggestions – Classical Conditioning | -0.177 (0.151) | .243 |
| Verbal Suggestions – Observational Learning | 0.272 (0.149) | .071 |
| Classical Conditioning – Observational Learning | 0.449 (0.128) | < .001 |

Results for the pairwise comparisons of the perceived influence between placebo learning mechanisms averaged across both groups. The estimated marginal means for every mechanism was subtracted from the other and this yielded the mean difference. Significance levels were tested with t-tests. SE = standard error of the mean.

**Table 3. Difference in acceptability of placebo-based strategies *between* groups**

| **Groups** | **Healthy controls to patients comparison** | |
| --- | --- | --- |
|  | Mean Difference (SE) | Significance level |
| General | 1.076 (0.386) | .006 |
| Closed-label | 1.342 (0.442) | .003 |
| Open-label | 1.076 (0.374) | .005 |
| Treatment-enhancing | 1.519 (0.373) | < .001 |
| Dose-extending | 0.709 (0.359) | .050 |

Results for the pairwise comparisons of the acceptability levels per placebo-based strategy between groups of patients and healthy controls. The estimated marginal means for every strategy was subtracted from the other and this yielded the mean difference. Significance levels were tested with t-tests. SE = standard error of the mean.

**Table 4. Difference in acceptability of placebo-based strategies within groups**

| **Groups** | **Patients** | | **Healthy controls** | |
| --- | --- | --- | --- | --- |
|  | MD (SE) | Significance level | MD (SE) | Significance level |
| General – Closed-Label | 1.646 (0.238) | < .001 | 1.380 (0.238) | < .001 |
| General – Open-Label | 0.063 (0.343) | .854 | 0.063 (0.343) | .854 |
| General – Enhancing | -0.759 (0.221) | < .001 | -1.203 (0.221) | < .001 |
| General – Dose-extending | -0.975 (0.229) | < .001 | -0.608 (0.229) | .009 |
| Closed-Label – Open-Label | -1.582 (0.444) | < .001 | -1.316 (0.444) | .004 |
| Closed-Label – Enhancing | -2.405 (0.310) | < .001 | -2.582 (0.310) | < .001 |
| Closed-Label – Dose-extending | -2.620 (0.302) | < .001 | -1.987 (0.302) | < .001 |
| Open-Label – Enhancing | -0.823 (0.303) | .007 | -1.266 (0.303) | < .001 |
| Open-Label – Dose-extending | -1.038 (0.337) | .002 | -0.671 (0.337) | .048 |
| Enhancing – Dose-extending | -0.215 (0.178) | .230 | 0.595 (0.178) | .001 |

Results for the pairwise comparisons of the acceptability levels for placebo-based strategies within groups of patients and healthy controls. The estimated marginal means for every strategy was subtracted from the other and this yielded the mean difference. Significance levels were tested with t-tests. SE = standard error of the mean.

**Table 5. Difference in acceptability of placebo-based strategies per symptom category *across* groups**

| **Groups** | **Pairwise comparisons** |  |
| --- | --- | --- |
|  | Mean Difference (SE) | Significance level |
| Psychological – Acute pain | 0.791 (0.203) | < .001 |
| Psychological – Chronic pain | 0.342 (0.175) | .053 |
| Psychological – Insomnia | -0.310 (0.150) | .041 |
| Acute pain – Chronic pain | -0.449 (0.167) | .008 |
| Acute pain – Insomnia | -1.101 (0.165) | < .001 |
| Chronic pain – Insomnia | -0.652 (0.131) | < .001 |

Results for the pairwise comparisons of the acceptability levels between symptom categories averaged across both groups. The estimated marginal means for every category was subtracted from the other and this yielded the mean difference. Significance levels were tested with t-tests. SE = standard error of the mean.

**Table 6. Difference in perceived effectiveness of placebo-based strategies *across* groups**

| **Groups** | **Pairwise comparisons** |  |
| --- | --- | --- |
|  | Mean Difference (SE) | Significance level |
| General – Closed-Label | -0.671 (0.086) | < .001 |
| General – Open-Label | 1.766 (0.191) | < .001 |
| General – Enhancing | -0.671 (0.122) | < .001 |
| General – Dose-extending | 0.158 (0.138) | .255 |
| Closed-Label – Open-Label | 2.437 (0.221) | < .001 |
| Closed-Label – Enhancing | 0.000 (0.122) | 1.000 |
| Closed-Label – Dose-extending | 0.829 (0.146) | < .001 |
| Open-Label – Enhancing | -2.437 (0.192) | < .001 |
| Open-Label – Dose-extending | -1.608 (0.187) | < .001 |
| Enhancing – Dose-extending | 0.829 (0.111) | < .001 |

Results for the pairwise comparisons of the perceived effectiveness between placebo-based strategies averaged across both groups. The estimated marginal means for every strategy was subtracted from the other and this yielded the mean difference. Significance levels were tested with t-tests. SE = standard error of the mean.

**Table 7. Exploratory analysis: Association between perceived effectiveness and acceptability of placebo-based strategies**

Multilevel linear mixed model of perceived effectiveness on acceptability.

| Fixed effect | Regression coefficient | | SE | *t* -value | *significance level* |
| --- | --- | --- | --- | --- | --- |
| Intercept | 3.895 | 0.396 | | 9.823 | < .001 |
| [Strategy=1] | -1.918 | 0.552 | | -3.472 | < .001 |
| [Strategy=2] | -4.176 | 0.556 | | -7.511 | < .001 |
| [Strategy=3] | 0.363 | 0.464 | | 0.782 | .434 |
| [Strategy=4] | -1.777 | 0.555 | | -3.201 | .001 |
| [Strategy=5] | 0^b^ | 0 | | . | . |
| Effectiveness | 0.555 | 0.067 | | 8.301 | < .001 |
| [Strategy=1] * Effectiveness | 0.185 | 0.093 | | 1.984 | .048 |
| [Strategy=2] * Effectiveness | 0.224 | 0.088 | | 2.540 | .011 |
| [Strategy=3] * Effectiveness | -0.082 | 0.089 | | -0.930 | .353 |
| [Strategy=4] * Effectiveness | 0.239 | 0.088 | | 2.715 | .007 |
| [Strategy=5] * Effectiveness | 0^b^ | 0 | | . | . |

Results for the exploratory analysis predicting the role of perceived effectiveness on acceptability of placebo-based strategies. Strategy 1 = general strategies, strategy 2 = closed-label strategies, strategy 3 = open-label strategies, strategy 4 = treatment-enhancing strategies, and strategy 5 = dose-extending strategies (reference group). The overall predictive relationship between perceived effectiveness and acceptability is shown under the heading “Effectiveness”. Significance was tested with t-tests. SE = standard error of the mean.

**Table 8.** **Placebo knowledge quiz outcomes per group**

| **Groups** | **Patients with FMS** | | **Healthy Controls** | |
| --- | --- | --- | --- | --- |
| Quiz items | N correct (%) | N incorrect (%) | N correct (%) | N incorrect (%) |
| 1 | 71 (89.9) | 8 (10.1) | 71 (89.9) | 8 (10.1) |
| 2 | 37 (46.8) | 42 (53.2) | 34 (43.0) | 45 (57.0) |
| 3 | 71 (89.9) | 8 (10.1) | 78 (98.7) | 1 (1.3) |
| 4 | 44 (55.7) | 35 (44.3) | 55 (69.6) | 24 (30.4) |
| 5 | 53 (67.1) | 26 (32.9) | 50 (63.3) | 29 (36.7) |
| 6 | 67 (84.8) | 12 (15.2) | 72 (91.1) | 7 (8.9) |
| 7 | 71 (89.9) | 8 (10.1) | 74 (93.7) | 5 (6.3) |

Results of the placebo quiz for every study group. The number of correctly or incorrectly scored answers per item is provided along with the percentages. FMS = fibromyalgia syndrome.

**Table 9. Exploratory analysis: Association between placebo knowledge and acceptability of placebo-based strategies.**

| **Linear Mixed Model Type III tests** | | | | |
| --- | --- | --- | --- | --- |
| Source | Numerator df | Denominator df | F-value | *p*-value |
| Intercept | 1 | ,000 | 60,995 | <,001 |
| Groups | 1 | 154,635 | 15,672 | <,001 |
| Strategy | 4 | 156,000 | 43,513 | <,001 |
| Interaction of Groups * Strategy | 4 | 156,000 | 2,954 | ,022 |
| Placebo Knowledge | 1 | 154,999 | 4,801 | ,030 |
| a. Dependent Variable: Acceptability | | | | |

Results for the exploratory analysis predicting the role of knowledge on acceptability when the effect of the placebo-based strategies is corrected for, i.e., their variance is included in the model.

**Table 10. Exploratory analysis: Association between placebo knowledge and perceived effectiveness of placebo-based strategies.**

| **Linear Mixed Model type III tests** | | | | |
| --- | --- | --- | --- | --- |
| Source | Numerator df | Denominator df | F-value | *p-*value |
| Intercept | 1 | 155,935 | 44,833 | <,001 |
| Groups | 1 | 155,146 | 10,680 | ,001 |
| Strategy | 4 | 156 | 46,029 | <,001 |
| Interaction of Groups * Strategy | 4 | 156,000 | 1,363 | ,249 |
| Placebo knowledge | 1 | 155,000 | 11,735 | <,001 |
| a. Dependent Variable: Perceived Effectiveness | | | | |

Results for the exploratory analysis predicting the role of knowledge on perceived effectiveness when the effect of the placebo-based strategies is corrected for, i.e., their variance is included in the model.

| Groups | **Males (N = 24)** | **Females (N = 75)** |  | |
| --- | --- | --- | --- | --- |
|  |  |  |  |  |
| Outcomes | Mean (SD) | Mean (SD) | *Main effect between groups* | |
| *Perceived influence of learning on pain relief (0-10 NRS)* |  |  | *F(1,97) = 0.251,*  *p* = .618, $\eta_{p}^{2}$= .003 | |
| Verbal Suggestion | 5.47 (2.36) | 5.76 (2.54) |  |  |
| Classical Conditioning | 5.49 (2.25) | 6.09 (2.54) |  |  |
| Observational Learning | 5.14 (2.23) | 5.54 (2.57) |  |  |
| *Main effect across groups* | *F*(2,192) = 4.692, *p* = .010, $\eta_{p}^{2}$= .05 | |  |  |
|  |  |  |  |  |
| *Acceptability of Strategies (0-10 NRS)* |  |  | *F(1,97) = 0.003,*  *p* = .953, $\eta_{p}^{2}$= .000 | |
| General | 6.75 (2.54) | 6.63 (2.14) |  |  |
| Closed-Label | 5.46 (2.93) | 5.21 (2.53) |  |  |
| Open-Label | 6.50 (3.12) | 6.68 (2.21) |  |  |
| Treatment-enhancing | 7.33 (2.67) | 7.88 (2.01) |  |  |
| Dose-extending | 7.50 (2.15) | 7.25 (2.14) |  |  |
| *Main effect across groups* | *F*(2.38, 230.95) = 2.947, *p* = 0.046, $\eta_{p}^{2}$= 0.03 | |  |  |
|  |  |  |  |  |
| *Acceptability in Symptoms (0-10 NRS)* |  |  | *F(1,96) = 0.522,*  *p* = .472, $\eta_{p}^{2}$= .005 | |
| Acute Pain | 4.92 (3.01) | 5.65 (2.66) |  |  |
| Chronic Pain | 6.21 (2.65) | 5.87 (2.64) |  |  |
| Psychological | 7.50 (2.09) | 6.17 (2.66) |  |  |
| Insomnia | 7.46 (2.19) | 6.80 (2.26) | *Interaction effect:* | |
| *Main effect across groups* | *F*(3, 291) = 21.346, *p* < .001, $\eta_{p}^{2}$= .18 | | *F*(2.77, 265.72) = 5.719, *p* = .001, $\eta_{p}^{2}$= .056 | |
| *Perceived Effectiveness of Strategies (0-10 NRS)* |  |  | *F(1,97) = 0.025,*  *p* = .874, $\eta_{p}^{2}$= .000 | |
| General | 6.38 (1.64) | 6.00 (1.78) |  |  |
| Closed-Label | 7.08 (2.36) | 6.79 (1.90) |  |  |
| Open-Label | 3.83 (2.60) | 4.16 (2.09) |  |  |
| Treatment-enhancing | 6.63 (1.72) | 6.89 (1.69) |  |  |
| Dose-extending | 6.04 (1.94) | 5.84 (2.10) |  |  |
| *Main effect across groups* | *F*(2.39, 232.19) = 53.537, *p* < .001, $\eta_{p}^{2}$= .36 | |  |  |
|  |  |  |  |  |
| Placebo knowledge (ratio of correct answers)* | 0.86 (0.14) | 0.86 (0.14) | Mann-Whitney U = 750,5, *p* = .205. | |

**Table 11. Exploratory analyses between men and women (healthy respondents):**

Results from the exploratory analyses between all healthy men and women. Essentially, all analyses from the primary and secondary analyses were re-run, but, this time, the groups were either healthy males or healthy females. The difference in groups for every individual level of an outcome (e.g., perceived influence of verbal suggestions between groups) was compared when the interaction effect for that model showed significance. Consecutively, the difference in levels of an outcome were compared within a group when this same interaction effect was significant (e.g., difference in extent of acceptability for every placebo-based strategy in healthy adults). For the placebo knowledge, descriptives were reported as median and IQR, whilst non-parametric testing was done with a Mann-Whitney test.

NRS = Numeric Rating Scale; SD = standard deviation.

**Table 12. Difference in perceived influence of placebo learning mechanisms *across* sexes**

| **Learning mechanisms** | **Pairwise comparisons** | |
| --- | --- | --- |
|  | Mean Difference (SE) | Significance level |
| Verbal Suggestion – Classical Conditioning | -0.222 (0.194) | .256 |
| Verbal Suggestion – Observational Learning | 0.350 (0.191) | .07 |
| Classical Conditioning – Observational Learning | 0.572 (0.179) | .002 |

Results for the pairwise comparisons of the perceived influence between placebo learning mechanisms averaged across sex. The estimated marginal means for every mechanism was subtracted from the other and this yielded the mean difference. Significance levels were tested with t-tests. SE = standard error of the mean.

**Table 13. Difference in acceptability of placebo-based strategies *across* sexes**

| **Groups** | **Pairwise comparisons** | |
| --- | --- | --- |
|  | Mean Difference (SE) | Significance level |
| General – Closed-Label | 1.353 (0.244) | < .001 |
| General – Open-Label | 0.098 (0.362) | .786 |
| General – Enhancing | -0.918 (0.216) | < .001 |
| General – Dose-extending | -0.688 (0.241) | .005 |
| Closed-Label – Open-Label | -1.254 (0.470) | .009 |
| Closed-Label – Enhancing | -2.271 (0.272) | < .001 |
| Closed-Label – Dose-extending | -2.041 (0.297) | < .001 |
| Open-Label – Enhancing | -1.017 (0.354) | .005 |
| Open-Label – Dose-extending | -0.787 (0.369) | .036 |
| Enhancing – Dose-extending | 0.230 (0.190) | .229 |

Results for the pairwise comparisons of the acceptability levels between placebo-based strategies averaged across sex. The estimated marginal means for every strategy was subtracted from the other and this yielded the mean difference. Significance levels were tested with t-tests. SE = standard error of the mean.

**Table 14. Difference in acceptability of placebo-based strategies per symptom category *between* sexes**

| **Symptom categories** | **Male to female comparison** | |
| --- | --- | --- |
|  | Mean Difference (SE) | Significance level |
| Psychological | 1.327 (0.595) | .028 |
| Acute pain | -0.737 (0.644) | .255 |
| Chronic pain | 0.342 (0.620) | .583 |
| Insomnia | 0.658 (0.526) | .214 |

Results for the pairwise comparisons of the acceptability levels per symptom category between sexes. The estimated marginal means for every sex per category was subtracted from the other and this yielded the mean difference. Significance levels were tested with t-tests. SE = standard error of the mean.

**Table 15. Difference in acceptability of placebo-based strategies per symptom category *across* sexes**

| **Groups** | **Males** | |  | **Females** |  | |
| --- | --- | --- | --- | --- | --- | --- |
|  | MD (SE) | Significance level | | MD (SE) | Significance level | |
| Psychological – Acute pain | 2.583 (0.505) | < 0.001 | | 0.520 (0.286) | .072 | |
| Psychological – Chronic pain | 1.292 (0.474) | 0.008 | | 0.307 (0.268) | .255 | |
| Psychological – Insomnia | 0.042 (0.407) | 0.919 | | -0.627 (0.230) | .008 |  |
| Acute pain – Chronic pain | -1.292 (0.412) | 0.002 | | -0.213 (0.233) | .363 |  |
| Acute pain – Insomnia | -2.542 (0.443) | < 0.001 | | -1.147 (0.251) | < .001 |  |
| Chronic pain – Insomnia | -1.250 (0.406) | 0.003 | | -0.933 (0.299) | < .001 |  |

Results for the pairwise comparisons of the acceptability levels between symptom categories within the two different sexes as the interaction effect was significant. The estimated marginal means for every category was subtracted from another and this yielded the mean difference. Significance levels were tested with t-tests. SE = standard error of the mean.

**Table 16. Difference in perceived effectiveness of placebo-based strategies *across* sexes**

| **Groups** | **Pairwise comparisons** |  | |
| --- | --- | --- | --- |
|  | Mean Difference (SE) | | Significance level |
| General – Closed-Label | -0.747 (0.118) | | < .001 |
| General – Open-Label | 2.191 (0.277) | | < .001 |
| General – Enhancing | -0.572 (0.175) | | .001 |
| General – Dose-extending | 0.247 (0.215) | | .254 |
| Closed-Label – Open-Label | 2.938 (0.315) | | < .001 |
| Closed-Label – Enhancing | 0.176 (0.170) | | .303 |
| Closed-Label – Dose-extending | 0.994 (0.236) | | < .001 |
| Open-Label – Enhancing | -2.763 (0.272) | | < .001 |
| Open-Label – Dose-extending | -1.944 (0.243) | | < .001 |
| Enhancing – Dose-extending | 0.818 (0.171) | | < .001 |

Results for the pairwise comparisons of the perceived effectiveness between placebo-based strategies averaged across sex. The estimated marginal means for every strategy was subtracted from the other and this yielded the mean difference. Significance levels were tested with t-tests. SE = standard error of the mean.

**Table 17. Exploratory analyses to evaluate the impact of the FSQ diagnostic criteria**

| Groups | **Patients with FMS (FSQ met)** | **Healthy Controls (FSQ not met)** |  | |
| --- | --- | --- | --- | --- |
|  | N = 73 | N = 77 |  |  |
| Outcomes | Mean (SD) | Mean (SD) | *Main effect between groups* | |
| *Perceived influence of learning on pain relief (0-10 NRS)* |  |  | *F*(1, 148) = 2.480, *p* = .117, $\eta_{p}^{2}$= .016 | |
| Verbal Suggestion | 5.33 (2.33) | 5.84 (2.48) |  |  |
| Classical Conditioning | 5.45 (2.27) | 6.13 (2.46) |  |  |
| Observational Learning | 5.16 (2.24) | 5.62 (2.52) |  |  |
| *Main effect across groups* | *F*(2, 296) = 3.539, *p* = .030, $\eta_{p}^{2}$= .02 | |  |  |
|  |  |  |  |  |
| *Acceptability of Strategies (0-10 NRS)* |  |  | *F*(1, 141) = 14.541, *p* < .001 | |
| General | 5.52 (2.62) | 6.65 (2.12) |  |  |
| Closed-Label | 3.66 (2.81) | 5.38 (2.49) |  |  |
| Open-Label | 5.84 (2.34) | 6.55 (2.20) |  |  |
| Treatment-enhancing | 6.34 (2.57) | 7.86 (1.98) |  |  |
| Dose-extending | 6.71 (2.21) | 7.29 (2.09) | *Interaction effect:* | |
| *Main effect across groups* | *F*(4, 141) = 42.584, *p* < .001 | | *F*(4, 141) = 4.446, *p* = .002 | |
| *Acceptability in Symptoms (0-10 NRS)* |  |  | *F*(1, 148) = 14.609, *p* < .001, $\eta_{p}^{2}$= .09 | |
| Acute Pain | 4.29 (2.71) | 5.68 (2.69) |  |  |
| Chronic Pain | 4.66 (3.02) | 6.07 (2.60) |  |  |
| Psychological | 5.07 (2.89) | 6.43 (2.50) |  |  |
| Insomnia | 5.19 (2.94) | 6.96 (2.13) |  |  |
| *Main effect across groups* | *F*(2.65, 392.80) = 15.608 , *p* < .001, $\eta_{p}^{2}$= .10 | |  |  |
|  |  |  |  |  |
| *Perceived Effectiveness of Strategies (0-10 NRS)* |  |  | *F*(1, 141) = 12.979, *p* < .001 | |
| General | 5.19 (2.03) | 6.12 (1.63) |  |  |
| Closed-Label | 5.59 (2.40) | 6.94 (1.73) |  |  |
| Open-Label | 3.51 (2.35) | 4.17 (2.13) |  |  |
| Treatment-enhancing | 5.58 (2.36) | 6.94 (1.66) |  |  |
| Dose-extending | 4.92 (2.20) | 5.88 (2.08) | *Interaction effect:*  *F*(4, 141) = 2.455, *p* = .049 | |
| *Main effect across groups* | *F*(4, 141) = 40.787 , *p* < .001 | |  |  |
| Placebo knowledge *(ratio of correct answers)** | 0.86 (0.14) | 0.86 (0.14) | Mann-Whitney U = 2700.5, *p* = .669. | |

Results from the exploratory analyses between patients meeting the FSQ diagnostic criteria and healthy controls not meeting the FSQ diagnostic criteria. Essentially, all analyses from the primary and secondary analyses were re-run, but, this time, the study groups either met the FSQ diagnostic criteria (when patient) or not (when healthy control). The difference in groups for every individual level of an outcome (e.g., perceived influence of verbal suggestions between groups) was compared when the interaction effect for that model showed significance. Consecutively, the difference in levels of an outcome were compared within a group when the same interaction effect was significant (e.g., difference in extent of acceptability for every strategy in healthy adults). For the placebo knowledge, descriptives were reported as median and IQR, whilst non-parametric testing was done with a Mann-Whitney test.

**Table 18.** **Difference in perceived influence of placebo learning mechanisms *across* groups**

| **Learning mechanisms** | **Pairwise comparisons** | |
| --- | --- | --- |
|  | Mean Difference (SE) | Significance level |
| Verbal Suggestion – Classical Conditioning | -0.205 (0.155) | .190 |
| Verbal Suggestion – Observational Learning | 0.193 (0.154) | .214 |
| Classical Conditioning – Observational Learning | 0.397 (0.138) | .004 |

Results for the pairwise comparisons of the perceived influence between learning mechanisms averaged across groups. The estimated marginal means for every mechanism was subtracted from the other and this yielded the mean difference. Significance levels were tested with t-tests. SE = standard error of the mean.

**Table 19. Difference in acceptability of placebo-based strategies *between* groups**

| **Groups** | **Healthy controls (FSQ -) to patients (FSQ+) comparison** | |
| --- | --- | --- |
|  | Mean Difference (SE) | Significance level |
| General | 1.119 (0.404) | .006 |
| Closed-label | 1.619 (0.445) | < .001 |
| Open-label | 0.848 (0.381) | .028 |
| Treatment-enhancing | 1.494 (0.389) | < .001 |
| Dose-extending | 0.543 (0.366) | .140 |

Results for the pairwise comparisons of the acceptability levels per placebo-based strategy between groups of patients meeting the FSQ criteria and healthy controls not meeting these. The estimated marginal means for every strategy was subtracted from the other and this yielded the mean difference. Significance levels were tested with t-tests. SE = standard error of the mean.

**Table 20. Difference in acceptability of placebo-based strategies within groups**

| **Groups** | **Patients with FSQ** | | **Healthy controls without FSQ** | |
| --- | --- | --- | --- | --- |
|  | MD (SE) | Significance level | MD (SE) | Significance level |
| General – Closed-Label | 1.773 (0.255) | < .001 | 1.273 (0.236) | < .001 |
| General – Open-Label | -0.167 (0.351) | .635 | 0.104 (0.325) | .749 |
| General – Enhancing | -0.833 (0.232) | < .001 | -1.208 (0.215) | < .001 |
| General – Dose-extending | -1.212 (0.239) | < .001 | -0.636 (0.222) | .005 |
| Closed-Label – Open-Label | -1.939 (0.450) | < .001 | -1.169 (0.417) | .006 |
| Closed-Label – Enhancing | -2.606 (0.326) | < .001 | -2.481 (0.302) | < .001 |
| Closed-Label – Dose-extending | -2.985 (0.315) | < .001 | -1.909 (0.292) | < .001 |
| Open-Label – Enhancing | -0.667 (0.316) | .036 | -1.312 (0.292) | < .001 |
| Open-Label – Dose-extending | -1.045 (0.343) | .003 | -0.740 (0.317) | .021 |
| Enhancing – Dose-extending | -0.379 (0.188) | .046 | -0.571 (0.174) | .001 |

Results for the pairwise comparisons of the acceptability levels for placebo-based strategies within groups of patients meeting the FSQ criteria and healthy controls not meeting these. The estimated marginal means for every strategy was subtracted from the other and this yielded the mean difference. Significance levels were tested with t-tests. SE = standard error of the mean.

**Table 21. Difference in acceptability per symptom category *across* groups**

| **Groups** | **Pairwise comparisons between symptoms** | |
| --- | --- | --- |
|  | Mean Difference (SE) | Significance level |
| Psychological – Acute pain | 0.767 (0.205) | < .001 |
| Psychological – Chronic pain | 0.387 (0.174) | .028 |
| Psychological – Insomnia | -0.328 (0.157) | .039 |
| Acute pain – Chronic pain | -0.380 (0.174) | .03 |
| Acute pain – Insomnia | -1.095 (0.166) | < .001 |
| Chronic pain - Insomnia | -0.715 (0.134) | < .001 |

Results for the pairwise comparisons of the acceptability levels between symptom categories averaged across groups. The estimated marginal means for every category was subtracted from the other and this yielded the mean difference. Significance levels were tested with t-tests. SE = standard error of the mean.

**Table 22. Difference in perceived effectiveness of placebo-based strategies *between* groups**

| **Groups** | **Healthy controls (FSQ -) to patients (FSQ+) comparison** | |
| --- | --- | --- |
|  | Mean Difference (SE) | Significance level |
| General | 0.890 (0.309) | .005 |
| Closed-label | 1.314 (0.347) | < .001 |
| Open-label | 0.639 (0.371) | .087 |
| Treatment-enhancing | 1.405 (0.347) | < .001 |
| Dose-extending | 0.959 (0.366) | .010 |

Results for the pairwise comparisons of the perceived effectiveness levels per placebo-based strategy between groups of patients meeting the FSQ criteria and healthy controls not meeting these. The estimated marginal means for every strategy was subtracted from the other and this yielded the mean difference. Significance levels were tested with t-tests. SE = standard error of the mean.

**Table 23. Difference in perceived effectiveness of placebo-based strategies within groups**

| **Groups** | **Patients with FSQ** | | **Healthy controls without FSQ** | |
| --- | --- | --- | --- | --- |
|  | MD (SE) | Significance level | MD (SE) | Significance level |
| General – Closed-Label | -0.394 (0.130) | .003 | -0.818 (0.120) | < .001 |
| General – Open-Label | 1.697 (0.267) | < .001 | 1.948 (0.247) | < .001 |
| General – Enhancing | -0.303 (0.166) | .069 | -0.818 (0.153) | < .001 |
| General – Dose-extending | 0.303 (0.193) | .118 | 0.234 (0.178) | .192 |
| Closed-Label – Open-Label | 2.091 (0.320) | < .001 | 2.766 (0.296) | < .001 |
| Closed-Label – Enhancing | 0.091 (0.173) | .599 | 0.000 (0.160) | 1.000 |
| Closed-Label – Dose-extending | 0.697 (0.211) | .001 | 1.052 (0.195) | < .001 |
| Open-Label – Enhancing | -2.000 (0.286) | < .001 | -2.766 (0.265) | < .001 |
| Open-Label – Dose-extending | -1.394 (0.269) | < .001 | -1.714 (0.249) | < .001 |
| Enhancing – Dose-extending | -0.606 (0.169) | < .001 | 1.052 (0.156) | < .001 |

Results for the pairwise comparisons of the perceived effectiveness levels for placebo-based strategies within groups of patients meeting the FSQ criteria and healthy controls not meeting these. The estimated marginal means for every strategy was subtracted from the other and this yielded the mean difference. Significance levels were tested with t-tests. SE = standard error of the mean.
